# *ProQEXAFS*: a highly optimized parallelized rapid processing software for QEXAFS data

**DOI:** 10.1107/S1600577519017053

**Published:** 2020-02-07

**Authors:** Adam H. Clark, Jerick Imbao, Ronald Frahm, Maarten Nachtegaal

**Affiliations:** a Paul Scherrer Institut, Forschungsstrasse 111, CH-5232 Villigen, Switzerland; b Institut für Materialwissenschaften und Fachbereich Physik, Bergische Universität Wuppertal, Gaußstraße 20, 42097 Wuppertal, Germany

**Keywords:** X-ray absorption spectroscopy, QEXAFS, XAS analysis

## Abstract

The high temporal resolution in data acquisition, possible in the quick-scanning EXAFS (QEXAFS) mode of operation, provides new challenges in efficient data processing methods. Here a new approach is developed that combines an easy to use interactive graphical interface with highly optimized and fully parallelized Python-based routines for extracting, normalizing and interpolating oversampled time-resolved XAS spectra from a raw binary stream of data acquired during *operando* QEXAFS studies. The programs developed are freely available via a Github repository.

## Abstract 2

A high-throughput program for the processing of QEXAFS spectra is presented.

## 1. Introduction   

The implementation of quick (or continuous) scanning X-ray absorption spectroscopy (XAS) methods, yielding moderate temporal resolution in the second to minute regime at least for conventional transmission experiments, has become commonplace at synchrotron facilities worldwide in recent decades (Frahm, 1988 ▸; Prieto *et al.*, 1992 ▸; Dent *et al.*, 2009 ▸; Prestipino *et al.*, 2011 ▸; Tanida *et al.*, 2011 ▸; Briois *et al.*, 2016 ▸; Müller *et al.*, 2016 ▸). The temporal resolution gives rise to the possibility of following changes in the local coordination and oxidation state of the element of interest during dynamic processes or study of metal–organic complexes before substantial radiation damage. More recent developments in scanning monochromators allow for a temporal resolution on the order of tens of milliseconds through sinusoidal oscillation about a Bragg angle such as implemented at the SuperXAS beamline of the Swiss Light Source (Müller *et al.*, 2016 ▸). The advent of quick-scanning EXAFS (QEXAFS) methods yielding sub-second temporal resolution allows for the study of kinetic and thermodynamic driven processes and finds particular use in areas such as catalytic and electrochemical systems among other applications (Grunwaldt & Frenkel, 2009 ▸; Rochet *et al.*, 2016 ▸; Vogt *et al.*, 2018 ▸; Marberger *et al.*, 2018 ▸; Kim *et al.*, 2019 ▸; Gaur *et al.*, 2019 ▸).

X-ray absorption fine structure is fundamentally a modulation of the absorption coefficient of a material as a consequence of the self-interference of a photoelectron, released by the excitation of a core electron by the back-scattering from nearby neighbouring atoms. As such, XAS is a powerful method to probe the local coordination geometry of the element of interest. Typically, XAS experiments use ion chambers before (*I*_0_) and after (*I*_1_) a sample. The attenuation of the incident X-ray beam yields the absorption spectrum,
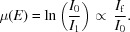
For dilute systems it is often necessary to measure in fluorescence mode, whereby the fluorescence photon flux (*I*_f_) is approximately proportional to the absorption coefficient (μ) of the element being probed. There are numerous examples in the literature of programs to evaluate the data collected during XAS experiments (Kuzmin, 1995 ▸; Ravel & Newville, 2005 ▸; Webb, 2005 ▸; Tenderholt *et al.*, 2007 ▸); however, to date these programs have not been designed for the processing of extremely large datasets typically acquired during fast QEXAFS experiments.

With the advancement of quick-scanning XAS methods with monochromator oscillation frequencies of the order of 1 Hz or faster, coupled with fast acquisition electronics with sampling frequencies of up to 2 MHz, extreme oversampling of an XAS spectrum is routinely acquired (Müller *et al.*, 2016 ▸). To control the monochromator and acquire data from several signals, a National Instruments PXIe-6366 multifunctional data acquisition board (DAQ) is used at the SuperXAS beamline of the Swiss Light Source and other facilities. The control of the monochromator angle is achieved through generation of sinusoidal control voltages to drive the torque motor which are outputted using digital-to-analogue converters (DACs) on the DAQ. The monochromator angular position is acquired using a quadrature angular encoder (angular resolution 0.00005°, 0.18 arcsec) and is recorded by the DAQ. The detector currents from either ion chambers or fluorescence detectors are amplified and the resulting voltages are digitized by analogue-to-digital converters (ADCs) of the DAQ with 16 bit resolution. The resulting highly oversampled XAS spectra allow for the removal of high-frequency noise, which originates from the data acquisition through filtering. Oversampling also provides the additional benefit wherein statistical measurement uncertainties can be calculated from the spread of values at any particular encoder value (energy step), which is otherwise unobtainable. This is due to the sampling frequency being in excess of the number of unique encoder values of the quadrature angular encoder.

As such, procedures for handling a large amount of data, including filtering and outputting statistical measurement uncertainties in a time-efficient manner, become highly desirable. The ability to access the acquired XAS data to guide further *operando* experiments requires the data processing to be performed on time scales of less than the data collection period. This allows for inspection of the data without significant delays. Here we discuss the application of parallelized processing procedures with highly optimized extraction, calibration, normalization and interpolation algorithms towards rapid data processing within an easy to use, freely available, Python-based graphical interface (Clark, 2019 ▸).

## 2. Graphical interface   

**Figure 1:**
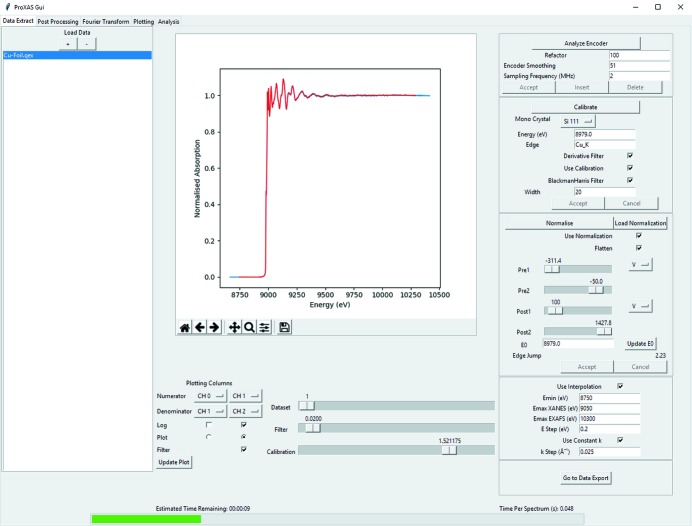
Graphical user interface demonstrating the usage for processing a Cu *K*-edge XAS spectrum.

The motivation for this software comes primarily from the need to increase the efficiency of the data processing pipeline for time-resolved and oversampled *operando* XAS spectroscopy studies. However, equally important, the ease of use for a larger user community was considered paramount. For this purpose, the use of Python-based programming was considered due to the readability and adaptability possibilities for a wide range of potential applications. The graphical user interface was developed using the well documented tk library and intended to be intuitive for novice user operation. Fig. 1 ▸ gives an overview of the graphical interface during batch processing.

**Figure 2:**
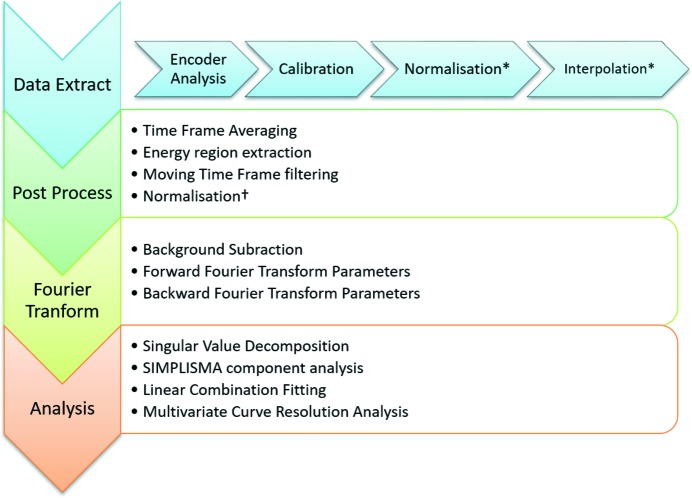
Flow chart outlining the standard data processing method. In the protocol for the data extraction tab, the normalization and interpolation steps are optional and denoted by *. The † symbol marking the normalization in the option post-processing utilities denotes that this is only possible in previously un-normalized data.

Typical user operation can be divided into four different processing steps: data extraction, post-processing, Fourier transformation and analysis. This manuscript is focused mainly on data extraction, highly optimized parallel processing algorithms and the application of Butterworth filtering for noise suppression. The flow chart given in Fig. 2 ▸ outlines the processing protocol and the additional post-processing routines and utilities provided with the software.

## 3. Data extraction, calibration and normalization   

During QEXAFS data collection, a binary stream of data is collected, storing channels for the monochromator encoder value, ion chamber voltages and photodiode voltages (for quick-fluorescence measurements) as columns. Further voltage channels can be additionally stored, representing, for example, the readout from a thermocouple or gas composition. At present, there are two available file formats. The first, the full raw data format, explicitly stores every sampled value at the full sampling rate. However, as a result of the finite resolution of the angular encoder and the high data sampling rates, for several samples, recorded during the sinusoidal movement of the monochromator, the recorded Bragg angle does not change. Thus a second file format, the ‘encoder reduced’ data format, aims to significantly compress the data files by storing the average signal values at a unique encoder value with additional columns for the statistical spread of each recorded channel. Choice of data format is made prior to data collection. In typical QEXAFS experiments there is a valuable advantage with regard to the resulting data size. By using the encoder reduced data format, significant compression of the data size compared with the full raw data format is achieved, on the order of 20-fold at 5 keV to 1000-fold at 24 keV, for scanning a Si(11) monochromator with an oscillation frequency of 1 Hz and an ADC sampling frequency of 2 MHz.

**Figure 3:**
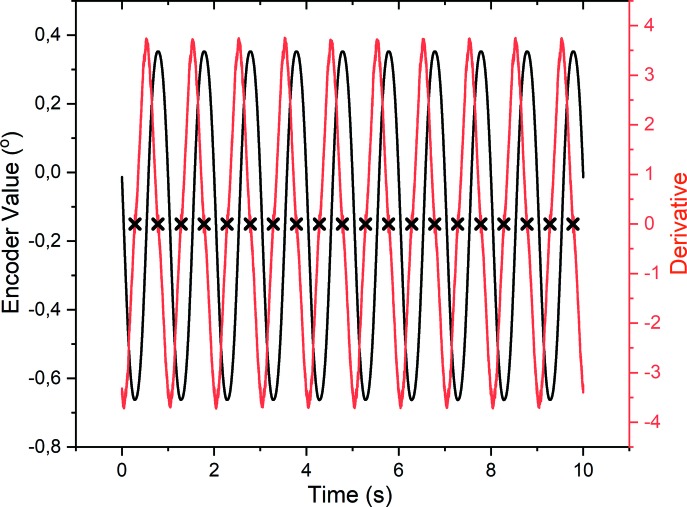
Plot showing the monochromator splitting using the zero crossing points of the angular encoder derivative. The encoder values are plotted as the black line with the red line giving the derivative and black crosses marking the extracted zero crossing points.

The first consideration for data extraction is to analyse the turning points of the monochromator of the monochromator encoder data and extract the direction of motion to yield single XAS spectra with an increasing or decreasing energy scale. This is achieved by finding the zero crossing points of the derivative of the encoder value derivative, as shown in Fig. 3 ▸.

Due to the large file sizes, typically many gigabytes, that are collected during *operando* QEXAFS measurements, the encoder data are buffered to only read in batches of 256 MB. This procedure allows large file reading to be parallelized and, as such, increases the efficiency of encoder analysis. Parallelization is performed in Python using a job queue system in coordination with the multiprocessing package in which individual buffered 256 MB segments of the datafile are processed in parallel by the number of logical processing cores available. Once fully analysed, the zero crossing points give the data point indices of the monochromator turning points.

**Figure 4:**
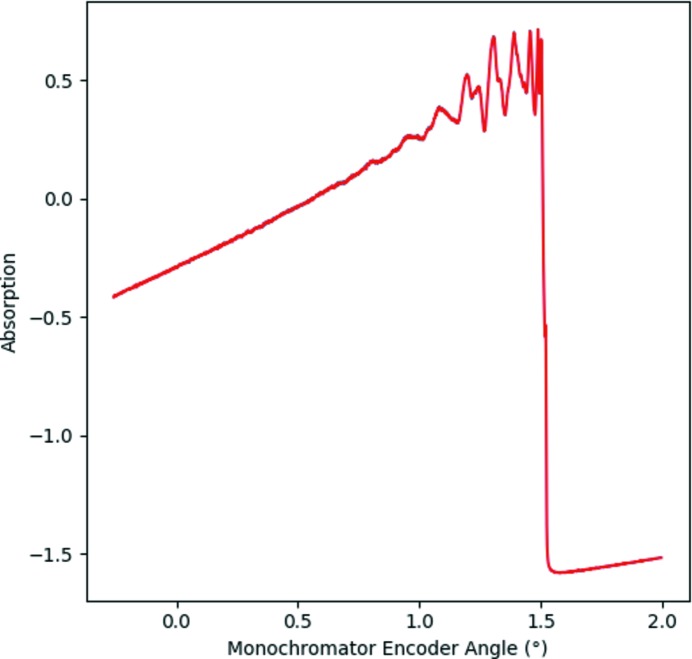
Plot showing a Cu *K*-edge XAS reference foil dataset between two monochromator turning points as a function of the angular encoder value. The offset between the angular encoder value and monochromator angle is refined during energy calibration from a known reference material.

**Figure 5:**
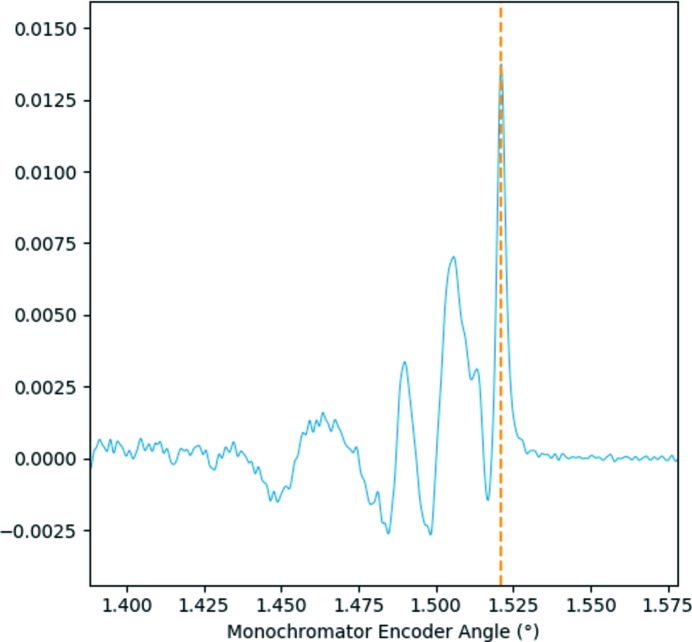
Plot showing the derivative of a Cu *K*-edge XAS reference foil (shown in blue) for calibration of an angle to a known energy. The calibration angle is indicated by the dashed orange line.

The data pertaining to a single XAS spectrum can thus be extracted by reading the data with indices between two monochromator turning points for each individual channel data for any point in time in the dataset. The first consideration after extraction of the data for a single spectrum is to remove any non-numerical values and, where appropriate, to average at unique encoder values. On very rare occasions, the ADC data yield a non-numerical value which must be removed for accurate data processing to be completed. As such, this process is performed automatically within the software. This averaging is necessary in the case where the data are recorded with the full sampling frequency for the raw data file format or if a defined maximum time for averaging is exceeded in encoder reduced file format. As such, a spectrum is acquired as a function of monochromator angle, as shown in Fig. 4 ▸. Calibration of the monochromator encoder angle is performed through consideration of the derivative of an XAS spectrum of a suitable reference material collected simultaneously with the XAS spectra. Typically, calibration of the monochromator angle to energy is achieved by setting the maximum of the derivative of a reference metal foil edge equal to a tabulated value. For example, the dashed line in the derivative of a Cu metal foil given in Fig. 5 ▸ indicates the angle for calibration to an energy of 8979 eV. Calibration for encoder value to energy is given by Bragg’s law,
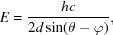
where the encoder value is given by θ and the offset between the encoder and the monochromator angle is given by φ and is determined from the selection of the calibration point. The value of 2*d* is the lattice spacing of the monochromator crystal, 6.27120 Å and 3.20267 Å for Si(111) and Si(311), respectively. The value of *hc* is given as 12398.42 eV Å.

**Figure 6:**
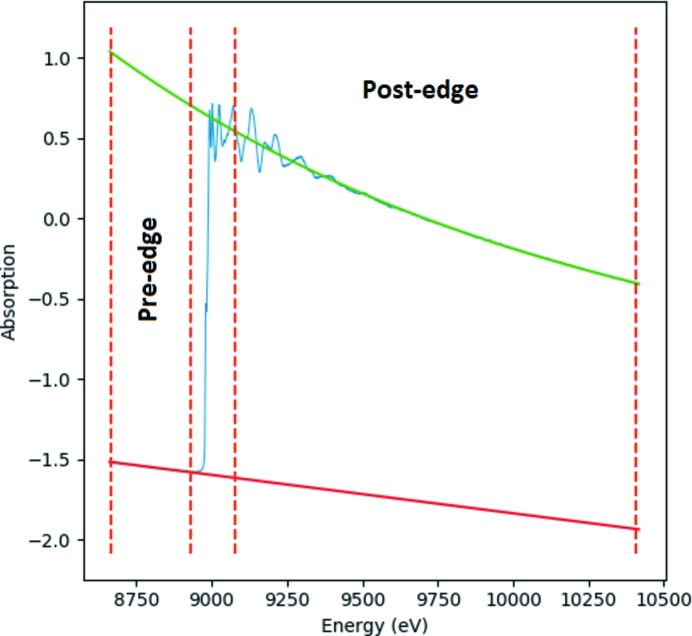
Example edge-step normalization showing the polynomial regression performed on the pre-edge (marked in red) and the post-edge (marked in green) regions. The XAS spectrum for a Cu foil collected at 1 Hz frequency is given in blue.

Normalization of XAS data is achieved through edge-step normalization to unity. For this purpose, a polynomial, typically first order, is fitted to the pre-edge region whereas the post-edge region is typically fitted by a third-order polynomial or a Victoreen function. An example of polynomial regression for the pre-edge and post-edge regions is given in Fig. 6 ▸. From fitting the pre-edge and post-edge functions, step normalization can be achieved using
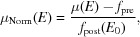
where μ(*E*) defines the raw spectrum with *f*_pre_ given as the extrapolated pre-edge polynomial function and *f*_post_ as the value of the extrapolated post-edge function at an edge energy of *E*_0_. For normalization, a choice of polynomial orders (zeroth, first, second, third and fourth) and Victoreen functions is available, with user-adjustable pre-edge and post-edge data ranges to be employed. The parameters chosen by the user for normalization are then applied automatically and identically to all spectra within a large dataset during the batch processing operation.

## 4. Data processing   

**Figure 7:**
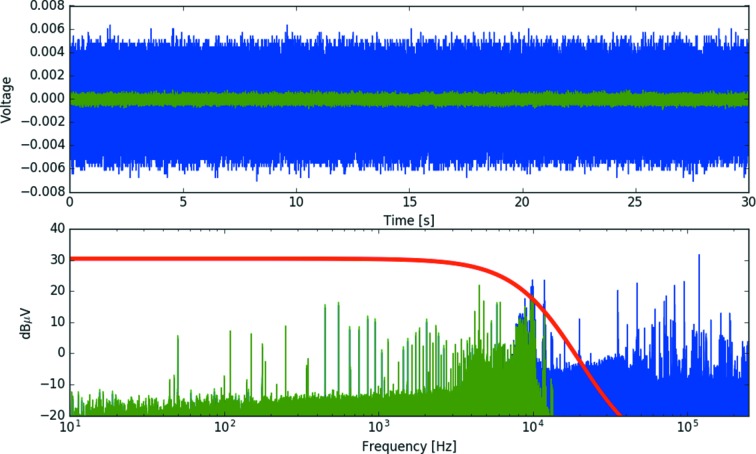
Noise sample collected without beam for estimation of the signal-to-noise ratio (top panel, blue); and the noise after application of the Butterworth filter with a 20 kHz cut-off frequency (top panel, green). Amplitude of noise frequencies (bottom panel, blue) and the effect of application of the Butterworth filter (bottom panel, green). The red line gives the scaled Butterworth gain factor.

Additional processing routines can be used to improve the quality of the acquired spectra and reduce the energy grid of the oversampled data to a practical energy grid. The high oversampling allows for Fourier filtering of high-frequency noise. Efficient Fourier filtering can be readily applied through the application of the Butterworth filter. The dependence of the gain set by the Butterworth filter is given by
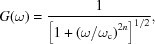
where ω is the frequency and ω_c_ is defined as the cut-off frequency. The order of the Butterworth filter is given by *n* and provides a smooth and continuous frequency filter with a tuneable cut-off frequency. As such, the Butterworth filter can be used to take advantage of the extreme oversampling achieved by the data acquisition system. To estimate the signal-to-noise ratio of the raw oversampled data, a 30 s sampling of noise (without beam) was acquired with amplifier gain of 10^6^ V A^−1^ with a standard deviation of 0.56 mV and a mean value of 0.065 mV. For a measurement performed at a 1 Hz monochromator oscillation frequency, the Butterworth filter is typically applied with a frequency cut-off of approximately 20 kHz and above, significantly above the highest frequency component of the XANES region. When applied to the sampling of noise without beam (Fig. 7 ▸), a standard deviation of 0.13 mV is achieved and a mean value of 0.065 mV. Thus a factor of 4.3 increase in the signal-to-noise ratio is achieved by applying the Butterworth filter. Signal-to-noise ratio is defined as the ratio of the mean signal and the standard deviation of that signal. Therefore it can be noted that, in the typical operating voltages of the amplifier outputs (∼0.5–10 V), this corresponds to a signal-to-noise ratio in excess of 3 × 10^3^. The raw (blue) and filtered (green) noise data are shown in the top panel of Fig. 7 ▸ with the bottom panel corresponding to the frequency components and scaled Butterworth gain, *G*(ω), in red.

**Figure 8:**
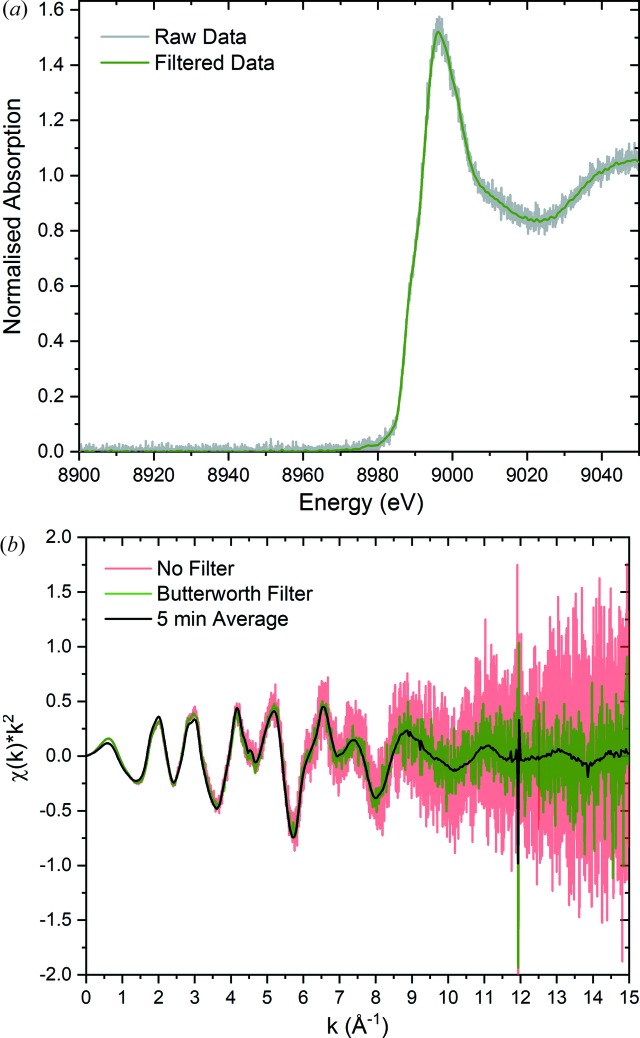
Application of a 20 kHz Butterworth filter on a Cu *K*-edge spectrum collected at 1 Hz. (Top) XANES region showing the raw data (grey) and filtered data (green). (Bottom) EXAFS region showing raw (red) and filtered 0.5 s (green) data and a 5 min averaged spectrum (black).

The application of the Butterworth filter to Cu *K*-edge spectra acquired at 1 Hz oscillation frequency on a CuO sample is shown in Fig. 8 ▸. Using a cut-off frequency typically on the order of 20 kHz does not damage the spectral features in either the XANES or EXAFS regions. Typically, the frequency component at which the leading edge of the XANES region rises is on the order of a few hundred Hz, when measured with a 1 keV data range using a 1 Hz monochromator oscillation frequency. The cut-off frequency is user defined and amendable. However, whilst the raw data file format allows specifically for a cut-off frequency to be defined due to storing data at the full sampling rate, this is not exactly possible for the encoder reduced file format with data averaging at unique encoder values. For this purpose, an initial estimate is performed internally as a guide through consideration of the energy step between calibrated encoder values at the edge position *E*_0_. However, the strength of the Butterworth filter, and whether it is applied, remains a user-adjustable parameter during data processing. Fig. 8 ▸ gives the density of encoder values as a function of energy. At 8979 eV, the angular resolution of the monochromator yields approximately 26 000 unique encoder values per keV. This is equivalent to a Δ*E* of 0.038 eV when approximating a linear angle–energy relation. For comparison, the intrinsic resolution of the Si(111) channel-cut monochromator (Ishikawa *et al.*, 2005 ▸) is given by
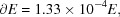
which yields an energy resolution of 1.2 eV at 8979 eV. Therefore the reduction of highly oversampled data onto a reduced energy grid yielding equivalent information content is highly desirable. For this purpose, radial basis function (RBF) interpolation is a powerful method. To decrease the number of data points and to allow compatibility with import into XAS fitting software packages such as *Demeter* (Ravel & Newville, 2005 ▸), highly optimized radial basis function interpolation has been developed with a user-defined energy grid. Implementation of the radial basis function interpolation was achieved using the *SciPy* Python package. Typically, an equally spread energy grid is applied over the XANES region whilst a user-defined constant *k*-grid (typically 0.025 Å^−1^) is used over the EXAFS region. Interpolation of data with a large number of data points is a computationally time-consuming process with the time taken to interpolate being approximately dependent on the square of the number of data points. To improve the efficiency of this process, localization of the radial basis function with windowed regions being interpolated has been implemented. Similar approaches have previously been applied primarily to reduce large 2D scattering datasets (Lazzaro & Montefusco, 2002 ▸; de Boer *et al.*, 2007 ▸; Xie & Liu, 2017 ▸).

**Figure 9:**
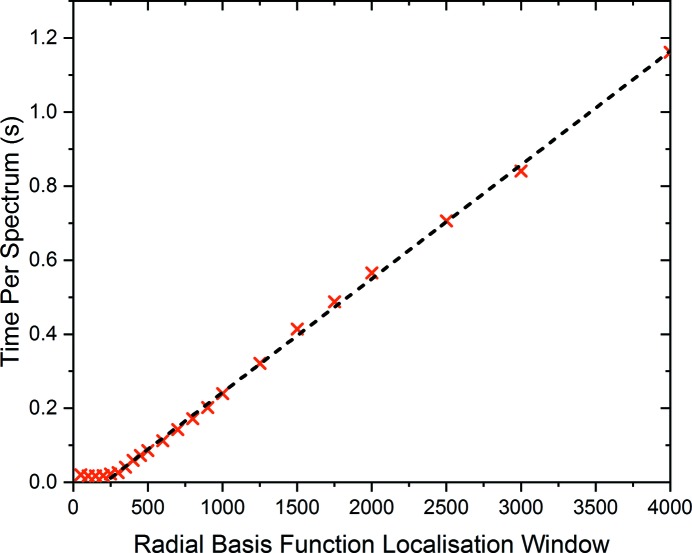
Time per spectrum for interpolation onto a user-defined energy grid as a function of radial basis function localization window. Measurements were performed using an 18 logical core CPU in parallel processing operation.

**Figure 10:**
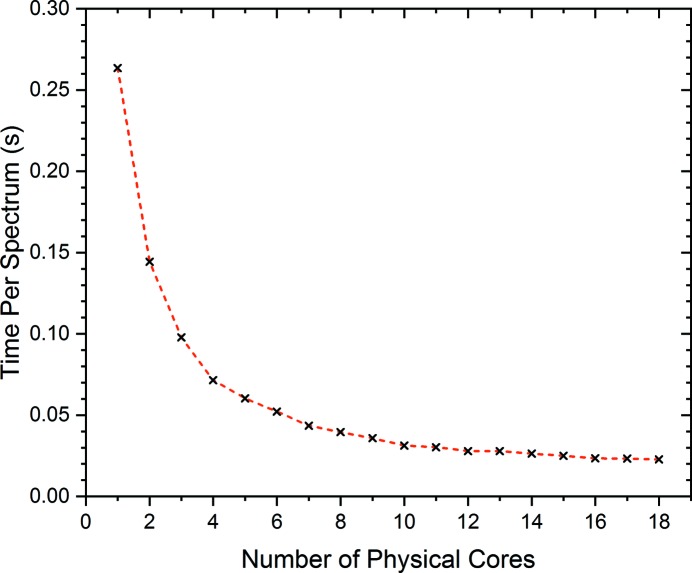
Processing time per spectrum as a function of the number of parallel processes spawned onto logical computational cores. The radial basis function localization window used was 250 data points.

The time taken per spectrum to interpolate as a function of window length has been explored and provides optimal performance with a window size of 250 data points, as shown in Fig. 9 ▸. Here a linear regression (shown in black) clearly demonstrates the efficiency achieved through localization of the radial basis function interpolation performed on data with 26 000 unique energy values. Efficient batch processing of QEXAFS spectra is achieved through parallelization by considering the indices of the turning point of the monochromator extracted during the encoder analysis. For this purpose, a queue system comprising the number of spectra to be processed is formed and jobs are spawned using the multiprocessing package in Python. The data-processing time per spectrum is shown in Fig. 10 ▸ as a function of the number of parallel processes spawned. Here, clear efficiency gains can be seen for the processing time per spectrum, although this is not a linear increase in efficiency. This is explained by reaching the i/o limit of the hardware for reading the data from the internal hard drives.

Batch processing of QEXAFS spectra applies the identical user-chosen parameters for calibration, normalization and interpolation to all spectra contained within a large dataset. The output data are given in ASCII format of an energy column followed by simple columns of number spectra. The ASCII format is chosen due to the ease of import into programs used for further data analysis. Batch processing can be performed with or without normalization to allow for normalization after averaging. Post-averaging normalization can result in a significant decrease in normalization error and is provided within a post-processing utility.

## 5. Post-processing utilities   

**Figure 11:**
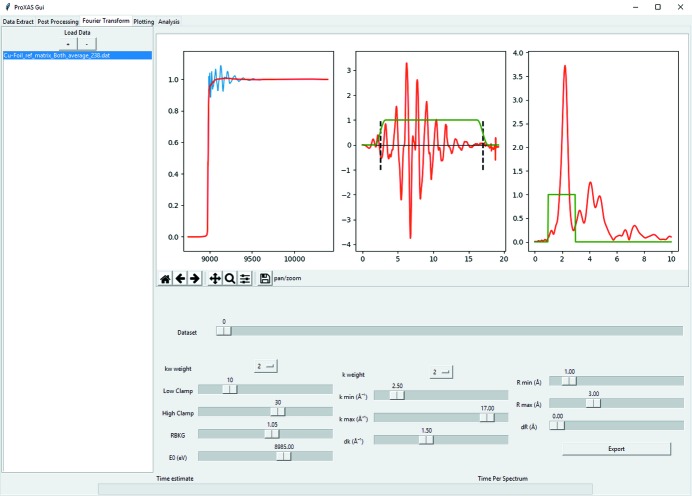
Graphical interface for interactive XAS background subtraction and Fourier transformation demonstrated for a Cu foil.

Within the software, there are a number of post-processing utilities. Data averaging is performed over a user-defined constant time period to improve the quality of spectra for either XANES or EXAFS analyses. Furthermore, it is possible to extract user-defined energy regions from the normalized data, for example, the XANES region or in data where there are multiple edges present in the region corresponding to the edge of interest. Through interfacing with Larch (Newville, 2013 ▸), batch background subtraction and Fourier transformation are possible. Simple user interaction with sliders allows for direct update of energy, *k*-space and *R*-space figures simultaneously to demonstrate the effect of changing a parameter in all spaces. An example of the user interface is shown in Fig. 11 ▸. Batch processing using identical user-chosen parameters for all spectra is performed iteratively with exported matrix files for energy, *k*-space and *R*-space individually that can be used for plotting or fitting in external programs such as *ARTEMIS* (Ravel & Newville, 2005 ▸).

## 6. Analysis utilities   

Analysis of the large datasets that are often collected in QEXAFS experiments can be a difficult task. Within the processing software some tools are provided for fast analysis of the data during beam time. These tools are intended to allow a quick view of the temporal evolution of a dataset to help in making efficient use of the beam time at SuperXAS. Giving an overview of the data, singular value decomposition (SVD) can be a powerful tool in estimating the number of species present in a dataset. To this end, a tool for truncated SVD has been implemented using the *SciPy* Python routines. When possible, linear combination fitting using known standards can be employed for fast analysis of the XANES region. However, it is not always possible to use known standards for these analyses; in such cases, the SIMPLISMA algorithm (Windig & Stephenson, 1992 ▸) provides a powerful method to give an estimate of the temporal evolution of the dataset by extracting the statistically most different spectra and performing a linear combination analysis. To further the analysis, multivariate curve resolution methods are currently being developed to provide a rapid view into large evolving datasets. The spectral components can be initialized using known standards or through extraction by application of the SIMPLISMA algorithm.

## 7. Concluding remarks   

Here we demonstrate the application of efficient parallelized algorithms for data reduction of highly oversampled QEXAFS spectra collected as a binary stream. The application of Butterworth noise filtering and optimized localized radial basis function interpolation is demonstrated, resulting in rapid data processing and significant improvement to the signal-to-noise ratio of an individual scan. Typical processing times on the order of 25 ms per spectrum can be readily achieved when using the original full raw data format. Significant raw data file compression has also been achieved, between 20- and 1000-fold with the implementation of a new encoder reduced data format with on-the-fly averaging at unique encoder values. An additional advantage of the new encoder reduced data format is revealed with processing times on the order of 7 ms per spectrum being readily achieved. With data processing per spectrum achieved on the order of milliseconds, immediate evaluation of data and data quality during QEXAFS experiments can be obtained and used to guide the effective use of experimental time. Broadly speaking, the methods described herein are transferable to other QEXAFS beamlines using the Wuppertal QEXAFS monochromator system without any modification. It is foreseen that the application of these processing methods could also be extended for use at other QEXAFS beamlines which collect highly oversampled XAS spectra.
